# An Estimation Formula for Resonance Frequency Using Sex and Height for Healthy Individuals and Patients with Incurable Cancers

**DOI:** 10.1007/s10484-023-09602-5

**Published:** 2023-09-13

**Authors:** Hideaki Hasuo, Keita Mori, Hiromichi Matsuoka, Hiroko Sakuma, Hideki Ishikawa

**Affiliations:** 1 Department of Psychosomatic Medicine, Kansai Medical University, Shinmachi 2-5-1, Hirakata, Osaka 573-1090 Japan; 2 Clinical Research Support Center, Shizuoka Cancer Center, Shizuoka, Japan; 3 Department of Psycho-Oncology, National Cancer Center Hospital, Tokyo, Japan; 4 Department of Molecular-Targeting Prevention, Kyoto Prefectural University of Medicine, Kyoto, Japan

**Keywords:** Resonance frequency, Heart rate variability, Estimation formula, Individual characteristics, Height, Sex

## Abstract

Resonance frequency breathing is a technique that involves breathing that maximizes heart rate variability. It is specific to individuals and is determined through a procedure taking approximately 30 min, using a procedure that is often best carried out at specialized medical institutions. This is a physical and time-consuming burden because of hospital visits and measurements, particularly for patients with cancer. Therefore it would be beneficial if a procedure can be found to determine resonance frequency from the patient’s physical characteristics, without the need for special assessment procedures. This exploratory cross-sectional study examined the correlation between individual characteristics and resonance frequency in healthy volunteers. Multiple regression analysis was performed with the measured resonance frequency as the target variable and individual characteristic parameters as explanatory variables. The study aims to build an estimation formula for resonance frequency with some of these parameters and assess its validity. In addition, the validity of the formula’s applicability to patients with incurable cancers is assessed. A total of 122 healthy volunteers and 32 patients with incurable cancers were recruited as participants. The median resonance frequency of 154 participants was six breaths per min. Sex and height were selected as explanatory variables associated with the measured resonance frequency in the volunteers. The estimation formula for resonance frequency using individual characteristics was *17.90—0.07* × *height* for men and *15.88—0.06* × *height* for women. Adjusted R-squared values were 0.55 for men and 0.47 for women. When the measured resonance frequency in patients with incurable cancers was six breaths per minute or less, the resonance frequency estimated by this formula was slightly larger than the measured ones. Information on individual characteristics, such as sex and height, which can be easily obtained, was useful to construct an estimation formula for resonance frequency.

## Introduction

Resonance frequency breathing (RFB), a respiration method that uses resonance frequency (or the optimal breathing rate) is a self-care technique to induce relaxation (Shaffer & Meehan, 2020). A protocol for heart rate variability biofeedback (HRV-BF) with RFB has been established, and some studies have indicated that HRV-BF with RFB is effective for blood pressure, mood stability, and sleep disturbance or depression after traumatic brain injury or for symptoms associated with bronchial asthma (Lehrer, 2021; Steffen et al., 2017; Vaschillo et al., 2006; Wearne et al., 2021). In oncology, RFB is reportedly a useful self-care technique to improve insomnia among patients with incurable cancers and their family caregivers, as well as those with decreased quality of life (Hasuo et al., 2019; Hasuo et al., 2020a; b). Early specialized palliative care contributes to improving the mood and quality of life by improving self care in patients with cancer (Greer et al., 2018).

Resonance frequency refers to the breathing rate at which heart rate variability (HRV) is maximized. HRV involves the fluctuation in time intervals between heartbeats measured from an electrocardiogram or pulse wave. RFB allows respiratory sinus arrhythmia to increase HRV, especially the low-frequency component (Bernardi et al., 2002; Lehrer, 2021; Vaschillo et al., 2002). The frequency range of the low-frequency component’s power is 0.04–0.15 Hz (Montano et al., 1994). Respiratory sinus arrhythmia is a phenomenon wherein the heart rate increases during inhalation and decreases during exhalation. Conversely, blood pressure has a fluctuation of approximately a 10-s cycle. When the respiratory cycle is consciously adjusted to the same frequency as that produced by blood pressure, the respiratory sinus arrhythmia produced by breathing becomes resonant with blood pressure. The phase relationship between respiratory sinus arrhythmia and blood pressure has been found to be 180° (Vaschillo et al., 2002). In recent years, reportedly, RFB also positively impacts the cardiovascular mechanisms of interoceptive awareness (Leganes-Fonteneau et al., 2021).

Resonance frequency is specific to individuals and is found to be 4.5–7 breaths per min (bpm) (Lehrer et al., 2013; Lehrer, 2021; Hasuo et al., 2019, 2020a, b; Schwerdtfeger et al., 2020); resonance effects are smaller when breathing at frequencies close to, but not exactly at, resonance frequency (Lehrer, 2021; Steffen et al., 2017). Resonance frequency calculation is conducted usually by specific professional medical organizations because monitoring is performed by a specialized HRV measurement system.

Resonance frequency is determined by specialists over a 20 min period based on synthetic judgments, such as power or amplitude value in the spectral peak of the HRV low-frequency component and waveform smoothing (Hinterberger et al., 2019; Lehrer, 2021). A simple method for calculating resonance frequency using only the spectral peak of the HRV low-frequency component has recently been developed (Sakakibara et al., 2020).

However, the fact that it can be performed only by specific professional medical organizations and takes approximately 30 min to measure has affected its widespread use and implementation. This is a physical and time-consuming burden in terms of hospital visits and measurements, particularly for patients with cancer. In addition to the frequent occurrence of physical function decline in patients with cancer, an association between its occurrence and prognosis has been reported (Ezzatvar et al., 2021). If the resonance frequency can be estimated simply by developing an estimation formula, it is expected to reduce physical and time-consuming burden for patients with cancer. However, to the best of our knowledge, there have been no reports on this.

Some reports that show a relationship between one’s height and resonance frequency. A first report on five healthy participants indicated that the higher the height, the more the resonance frequency may decrease (Shaffer & Meehan, 2020). A report on 32 asthmatic patients and 24 healthy individuals showed a correlation coefficient of − 0.55 between height and resonance frequency, indicating a moderate inverse correlation (Vaschillo et al., 2006). Furthermore, a report on 50 patients with incurable cancers with insomnia suggested a possible relationship between height, age, and resonance frequency, such as a correlation coefficient of − 0.59 (Hasuo et al., 2020b, 2021). Limitations of these studies existed owing to fewer participants, selection bias caused by patient background inhomogeneity, and information bias in which researchers knew the characteristics of the participants (Hasuo et al., 2020b, 2021; Shaffer & Meehan, 2020; Vaschillo et al., 2006;).

Therefore, we aimed to build an estimation formula for resonance frequency with some of the individual characteristic parameters.

## Methods

### Study Participants and Eligibility Criteria

Overall, 122 healthy volunteers (men and women, aged 20–85 years) were recruited. Healthy volunteers were defined as persons who were normal, without any significant medical conditions or history, and with no difficulty in daily living. Participants visited the university’s website and volunteer recruitment bulletin boards at Kansai Medical University and its hospital. The exclusion criteria were as follows: (1) currently taking medication or visiting medical facilities regularly, (2) having physical diseases, such as lung diseases, and (3) having experience with yoga and, breathing techniques. They were recruited to homogenize the number and sex of each generation.

Second, 32 patients with incurable cancers who underwent surgery at the Department of Palliative Care at Kansai Medical University Hospital (men and women, aged 20–85 years) were recruited. Exclusion criteria were as follows: (1) having respiratory-related symptoms (e.g., dyspnea, respiratory-induced cough, etc.), (2) having experience with yoga and, breathing techniques and (3) having mental disorders (e.g., mood disorder, cognitive impairment, etc.). Structured clinical interviews for mental disorders were conducted to exclude them. They were not recruited to homogenize the number and sex of each generation.

### Study Design

After conducting an exploratory cross-sectional study on individual characteristic parameters that correlated with the measured resonance frequency in healthy volunteers, we aimed to build an estimation formula to measure resonance frequency with these parameters and assess its validity. In addition, we assessed the validity of the formula’s applicability to patients with incurable cancers.

This study was approved by the Medical Ethics Committee of Kansai Medical University (reference number: 2021079) and was performed in accordance with the Declaration of Helsinki (as revised in 2013). Written informed consent was obtained from all study participants prior to the commencement of any study procedure. This study was conducted from January–April 2022.

### Measures

The investigator collected basic information from all participants, including age, sex, height, and weight. The investigator also collected additional information from participants with incurable cancers, including the Eastern Cooperative Oncology Group performance status, primary cancer sites, treatment options, and medications. Subsequently, the chest and abdominal circumferences were measured in all participants.

An HRV measurement device (myBeat WHS-1; Union Tool Co., Tokyo, Japan) was attached to the dedicated electrode pad. The pad was applied directly to each participant’s chest for the continuous measurement of HRV. During the measurement, the participant breathed at a pace of 5 bpm, 5.5 bpm, 6 bpm, 6.5 bpm, and 7 bpm for 3 min each in a seated position, using the Breath Pacer application (ProComp Infiniti™/BioGraph Infiniti, Thought Technology Ltd., Montreal, Canada) as a subsidiary.

Two other investigators (who stayed in another room to avoid facing the participants and being blinded to participants’ individual information) evaluated various spectra and waveforms of the participants’ HRV obtained with an HRV analysis software (RRI Analyzer2; Union Tool Co., Tokyo, Japan) via Bluetooth, and determined the optimal measured resonance frequency for the participants. The optimal measured resonance frequency was defined as the paced breathing rate with the highest low-frequency band power level in the HRV spectral analysis during each participant’s paced breathing and smooth waveform of HRV (Hinterberger et al., 2019; Lehrer, 2021).

### Sample Size Calculation

For this prospective study, a larger number of cases would result in a better estimation formula. Therefore, we recruited as many participants as possible during the study period. We considered 8:2 as the split method between the training and test sets when building an estimation formula (Fan et al., 2022; Song et al., 2011). The estimated number of healthy volunteers and patients with incurable cancers was 120 and 30, respectively. The resonance frequency (the primary endpoint) is a continuous wave. Multiple linear regression was used to create an estimation formula for resonance frequency. The overall numbers of participants required for the study was set at 150. As for sample size calculations, we examined them in terms of the rule of 10 and, subsequently, confirmed them based on the maximum number of variables that can be handled simultaneously.

### Statistical Analysis

Data were reported as mean with standard deviation, median with interquartile range, or frequency (%) with a 95% confidence interval. Data from healthy volunteers were used for training to create an estimation formula for resonance frequency (Armitage et al., 2010). Multiple regression analysis was performed using the measured resonance frequency, continuous data (as the target variable), and sex as categorical data, in addition to age, height, weight, chest circumference, and abdominal circumference, as candidate explanatory variables. First, univariate analyses were performed on resonance frequency and age, sex, height, body weight, chest circumference, and abdominal circumference (univariate in Table 2). Then, a multivariate analysis was performed on the resonance frequency, including sex, height, body weight, chest circumference and abdominal circumference. Model 1 presents the results. Specifically, sex and height were significant, so a multivariate analysis was performed on the resonance respiratory rate including only these two variables. Model 2 presents this result. After obtaining the results of Models 1 and 2, as presented in Table 2, we discussed how we would proceed with further analyses. Specifically, we discussed whether it would be possible to separate the formulas for male and female participants. Accordingly, we separated the formulas and created a regression equation (Model 3) to predict the resonance respiratory rate by height for male patients only. We created another regression equation (Model 3) for female patients, which was the model adopted as the result of the study. After each analysis, the results were shared among the authors to confirm that we adoptd a model that can estimate resonance frequencies by easily obtainable individual characteristics, rather than a model with high explanatory power and develop a formula for estimating the optimal resonance frequency.

To determine whether the created estimation formula for resonance frequency can also be applied to patients with incurable cancers, we obtained the predicted resonance frequencies obtained by this estimation formula and the actual measured resonance frequencies of the patients and subsequently prepared a box-and-whisker diagram to show the correlation between the two variables.

Statistical significance was set at p < 0.05. The R software (ver. 4.0.5) was used for the data analyses.

## Results

### Number of Study Participants and Individual Characteristics

All the recruited 122 healthy volunteers and 32 patients with incurable cancers completed the study (100%). Table 1 presents the demographic and clinical characteristics of the participants. Among patients with incurable cancers, 23 received chemotherapy and 9 received the best supportive care alone.

**Table 1:** Demographic and basic characteristics of the study participants

Variable	Healthy volunteers		Patients with incurable cancer	
Age (years), mean (SD)	46.5	(17.6)	68.4	(9.5)
Sex, male, n (%)	61	(50.0)	16	(50.0)
Height (cm), mean (SD)	163.9	(8.7)	159.1	(7.4)
Body weight (kg), mean (SD)	58.1	(9.8)	50.7	(9.5)
Chest circumference (cm), mean (SD)	87.5	(6.7)	84.8	(6.9)
Abdominal circumference (cm), mean (SD)	79.3	(9.8)	81.4	(9.8)
In-room oxygen saturation (%), median (IQR)	98.0	(97–99)	98	(97–99)
Resonant frequency (bpm), median (IQR)	6	(5.5–6.0)	6.25	(6.0–6.5)
Resonant frequency (bpm), n (%)				
5.0	12	(9.8)	0	(0.0)
5.5	21	(17.2)	2	(6.3)
6.0	34	(27.9)	14	(43.7)
6.5	26	(21.3)	9	(28.1)
7.0	29	(23.8)	7	(21.9)
Primary cancer site, n (%)				
Lung			4	(12.5)
Gastrointestinal			14	(43.7)
Breast			3	(9.3)
Liver, pancreas			2	(6.3)
Gynaecological			2	(6.3)
Urological			2	(6.3)
Head and neck			5	(15.6)
ECOG PS, n (%)				
0			3	(9.3)
1			7	(21.9)
2			15	(46.9)
3			7	(21.9)
4			0	(0.0)

*SD* standard deviation, *IQR* interquartile range; bpm, breaths per minute, *ECOG PS* Eastern Cooperative Oncology Group performance status

### Creation of an Estimation Formula for Resonance Frequency in Healthy Volunteers

Table 2 shows the multiple regression analysis of the measured resonance frequency as the target variable and other explanatory variables. Model 1 shows the results of the multiple regression analysis with all explanatory variables; Model 2 shows the results of the second analysis performed with variables that were significant in the first analysis; and Model 3 shows the results of the analysis using the ultimately selected estimation formula for resonance frequency. As a result of the regression analysis, point estimates of the regression coefficients for each variable, standard errors, upper and lower limits of the 95% confidence intervals, and p-values were calculated, and the adjusted R-squared for each model was also obtained. Adjusted R-squared were 0.58 for Model 1; 0.58 for Model 2; and 0.55 and 0.47 for men and women, respectively, in Model 3.

**Table 2:** Multiple regression analyses of measured resonance frequency as the target variable and the other explanatory variables

Model	Model 1							Model 2				Model 3					
Analysis	Univariate			Multivariate				Multivariate				Multivariate					
												Male			Female		
Variable	β	(95% CI)	P	β	(95% CI)	P	VIF	β	(95% CI)	P	VIF	β	(95% CI)	P	β	(95% CI)	P
Age	0.002	(− 0.004 to − 0.008)	0.516														
Sex	0.478	(0.265–0.692)	< 0.001	− 0.356	(− 0.568 to − 0.144)	0.001	2.05	− 0.278	(− 0.477 to − 0.079)	0.007	1.79						
Height	− 0.055	(− 0.064 to − 0.046)	< 0.001	− 0.066	(− 0.081 to − 0.051)	< 0.001	3.15	− 0.066	(− 0.077 to − 0.054	< 0.001	1.79	− 0.071	(− 0.087 to − 0.054)	< 0.001	− 0.06	(− 0.076 to − 0.044)	< 0.001
Body weight	− 0.034	(− 0.044 to − 0.024)	< 0.001	− 0.003	(− 0.022 to − 0.0168)	0.776	6.71										
Chest circumference	− 0.03	(− 0.047 to − 0.014)	< 0.001	0.007	(− 0.013 to − 0.028)	0.479	3.3										
Abdominal circumference	− 0.015	(− 0.026 to − 0.003) ()	0.013	− 0.01	(− 0.023 to − 0.002)	0.111	2.82										
Adjusted R-squared				0.5857				0.58				0.55			0.47		

*β* standardized partial regression coefficient, *CI* confidence interval

Finally, the estimation formula for the resonance frequency (Model 3) was as follows:

Males: 17.90 − 0.07 × height.

Females: 15.88 − 0.06 × height.

### Extrapolation of an Estimation Formula for Resonance Frequency in Healthy Volunteers to Patients with Incurable Cancers

**Fig. 1:**
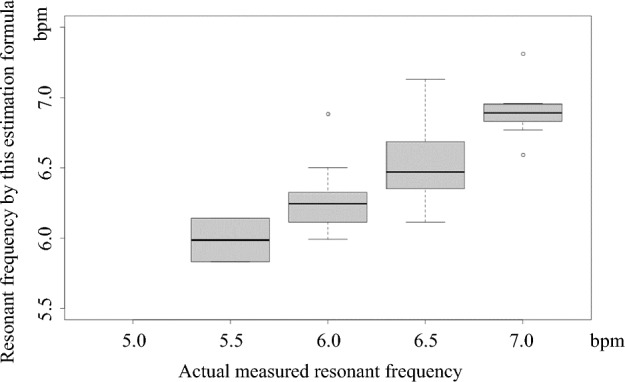
Box-whisker diagram showing the relationship between estimated resonance frequency and measured resonance frequency in patients with incurable cancers. *bpm, breaths per minute

In patients with incurable cancers, a box-and-whisker diagram showing the relationship between the final estimation formula for resonance frequency (Model 3) obtained from healthy volunteers and the actual resonance frequency measured by the HRV measurement device is shown in Fig. 1. When the actual measured resonance frequency in patients with incurable cancers was higher than 6 bpm, the resonance frequency estimated by this estimation equation approximated the actual measured ones. Contrarily when the actual measured resonance frequency was less than 6 bpm, the resonance frequency estimated by this estimation equation was slightly higher than the measured ones.

## Discussion

To the best of our knowledge, this is the first report on the establishment of an estimation formula for resonance frequency with individual characteristics.

In this study, notably, that height had the most significant correlation with resonance frequency among individual characteristics, which aligns with previous studies (Hasuo, et al., 2021, 2020b; Vaschillo, et al., 2006). This study had high reliability because of the relatively large number of participants, and fewer selection and information biases. The correlation between the two was very strong, and it was possible to build an estimation formula for resonance frequency centered on height. Sex was included in this estimation formula because it also slightly correlated with the partial correlation which considered height as a control variable. Resonance frequency was lower in men than in women, which aligns with previous studies (Hasuo, et al., 2021; Vaschillo, et al., 2006). We believe that height and sex are significant in that they are factors that change little over time and in terms of disease.

Compared with other parameters, participants’ height was significantly correlated with resonance frequency. To the best of our knowledge, no report explains the reason for this strong correlation between height and resonance frequency. The reasons for this correlation were not determined in this study and will need to be investigated from respiratory and circulatory dynamics in the future. Considering circulatory dynamics, one predictor of circulatory blood volume is height (Brown et al., 1962). It is related to the amount of time it takes for blood pressure to change after the baroreflex causes the heart rate to change. This is related to inertia in the blood supply.

Considering previous studies, weight and age showed a less significant correlation with resonance frequency (Hasuo, et al., 2021; Vaschillo, et al., 2006). Weight is difficult to use as a parameter because the weight loss percentage is high owing to cancer cachexia, which develops in 25% of patients with incurable cancers (Vagnildhaug et al., 2019). Adipose data has little blood supply. Thus, it does not contribute to resonance frequency. For age, caution is required because it is difficult to identify resonance frequency. In older adults, despite the exception of in-phase relationships, breathing reportedly stimulates baroreflex and generates high-amplitude heartbeat vibrations (Lehrer et al., 2020).

The second important aspect of this study is the successful establishment of an estimation formula for resonance frequency with individual characteristics. No correlation was found between the measured resonance frequency and age; moreover, a previous report on resonant frequency showed no changes before and after ten biofeedback sessions (Vaschillo et al., 2006). In other words, because it is identified by individuals (and remains unchanged), an estimation formula for the resonance frequency that can be created only from individual characteristics is significant. In addition, an estimation formula for resonance frequency constructed only from easily obtainable individual characteristics (such as height and sex) is also significant. The existence of this estimation formula may positively impact the widespread implementation of the resonance breathing method. Contrarily, the adjusted R-squared for Model 3 was not substantially large. The emphasis of this study was not to propose a highly explanatory model, but rather to show that resonance frequency can be estimated by easily obtainable individual characteristics.

In recent years, devices for measuring HRV have been developed. With this development, HRV-BF with RFB can now be performed at home (Hasuo et al., 2019, 2020a, b). However, as in our study of patients with incurable cancer (Hasuo et al., 2020b), RFB identification must be performed at the outpatient department of a professional medical organization. In a report on HRV-BF sessions with RFB in patients with incurable cancers with insomnia, participants were limited to those who could see a doctor regularly in a professional medical organization (Hasuo, Kanbara, Shizuma, et al., 2020). However, an estimation formula for resonance frequency made it possible to identify resonance frequency at home and contributed to the widespread use of HRV-BF with RFB in the primary region.

Based on the box-and-whisker diagram (Fig. 1), it is reasonable to extrapolate the estimation formula for the resonance frequency calculated in healthy volunteers to patients with incurable cancers. However, for patients with incurable cancers, whose measured resonance frequency was 6 bpm or less, the resonance frequency estimated by this formula was slightly higher than the measured frequency. This may be because of the small number of patients with incurable cancers enrolled in this study, especially those with measured resonance frequency of 6 bpm or less: none with a measured resonance frequency of 5 bpm, two with 5.5 bpm, and 14 with 6 bpm. In the future, larger patient population data will be required to extrapolate this estimation formula to patients with incurable cancers. In addition, information on pulmonary function, such as lung capacity and auxiliary respiratory muscle function, would be desirable. In an in-vivo study, cachexia, common in patients with incurable cancers, caused respiratory failure and loss of auxiliary respiratory muscle function, such as that of the diaphragm (Fields et al., 2019). However, there is no evidence to show that asthma severity is related to resonance frequency (Vaschillo et al., 2006), and whether pulmonary function influences an estimation formula for resonance frequency remains unclear.

This study had three limitations. First, the participants in this study underwent resonance frequency assessment for paced breathing at 5 bpm, 5.5 bpm, 6 bpm, 6.5 bpm, and 7 bpm, respectively. However, the standard protocol for identifying resonance frequency includes a rate of 4.5 bpm (Lehrer et al., 2013). Second, the study did not use the univariate p-value screening and multivariate analysis method commonly used in clinical articles. Although such a method is arbitrary, the study could suggest the possibility of creating an estimation formula that could predict resonance frequency from only simple variables. Third, from the perspective of dissemination and implementation, the explanatory variables were limited to information that was readily available and relatively stable. The adjusted R-squared value could have been significant if the explanatory variables included information on lung function, such as lung capacity and function of the ventilatory assist muscles, which were not evaluated in this study. However, there is no evidence that asthma severity is related to resonance frequency (Vaschillo et al, 2006), and it is unclear whether pulmonary function influences an estimation formula for resonance frequency. Finally, the number of patients with incurable cancers was low, which may have affected the extrapolation of the estimation formula for resonance frequency in healthy volunteers to patients with incurable cancers.
